# Development of an action programme tackling obesity-related behaviours in adolescents: a participatory system dynamics approach

**DOI:** 10.1186/s12961-024-01116-8

**Published:** 2024-03-01

**Authors:** Angie Luna Pinzon, Wilma Waterlander, Naomi de Pooter, Teatske Altenburg, Coosje Dijkstra, Helga Emke, Emma van den Eynde, Meredith L. Overman, Vincent Busch, Carry M. Renders, Jutka Halberstadt, Wilma Nusselder, Karen den Hertog, Mai Chinapaw, Arnoud Verhoeff, Karien Stronks

**Affiliations:** 1 Department of Public and Occupational Health, Amsterdam UMC Location University of Amsterdam, Meibergdreef 9, Amsterdam, The Netherlands; 2 Health Behaviors and Chronic Diseases, Amsterdam Public Health Research Institute, Amsterdam, The Netherlands; 3 Department of Public and Occupational Health, Amsterdam UMC Location Vrije Universiteit Amsterdam, De Boelelaan 1117, Amsterdam, The Netherlands; 4 Department of Health Sciences, Faculty of Science, Vrije Universiteit Amsterdam, 1081HV Amsterdam, The Netherlands; 5 Division of Endocrinology, Department of Pediatrics, Erasmus MC-Sophia Children’s Hospital, University Medical Centre Rotterdam, Rotterdam, The Netherlands; 6 Department of Health Promotion, NUTRIM School of Nutrition and Translational Research in Metabolism, Maastricht University, 6229ER Maastricht, The Netherlands; 7 Sarphati Amsterdam, Public Health Service (GGD), City of Amsterdam, Nieuwe Achtergracht 100, 1018WT Amsterdam, The Netherlands; 8 Department of Public Health, Erasmus MC, University Medical Center Rotterdam, 3015CN Rotterdam, The Netherlands; 9 Amsterdam Healthy Weight Approach, Public Health Service (GGD), City of Amsterdam, Nieuwe Achtergracht 100, 1018WT Amsterdam, The Netherlands; 10 Department of Sociology, University of Amsterdam, 1018WV Amsterdam, The Netherlands

**Keywords:** Overweight and obesity, Systems thinking, Whole-of-systems approaches, Complex systems, Adolescents

## Abstract

System dynamics approaches are increasingly addressing the complexity of public health problems such as childhood overweight and obesity. These approaches often use system mapping methods, such as the construction of causal loop diagrams, to gain an understanding of the system of interest. However, there is limited practical guidance on how such a system understanding can inform the development of an action programme that can facilitate systems changes. The Lifestyle Innovations Based on Youth Knowledge and Experience (LIKE) programme combines system dynamics and participatory action research to improve obesity-related behaviours, including diet, physical activity, sleep and sedentary behaviour, in 10–14-year-old adolescents in Amsterdam, the Netherlands. This paper illustrates how we used a previously obtained understanding of the system of obesity-related behaviours in adolescents to develop an action programme to facilitate systems changes. A team of evaluation researchers guided interdisciplinary action-groups throughout the process of identifying mechanisms, applying the Intervention Level Framework to identify leverage points and arriving at action ideas with aligning theories of change. The LIKE action programme consisted of 8 mechanisms, 9 leverage points and 14 action ideas which targeted the system’s structure and function within multiple subsystems. This illustrates the feasibility of developing actions targeting higher system levels within the confines of a research project timeframe when sufficient and dedicated effort in this process is invested. Furthermore, the system dynamics action programme presented in this study contributes towards the development and implementation of public health programmes that aim to facilitate systems changes in practice.

**Supplementary Information:**

The online version contains supplementary material available at 10.1186/s12961-024-01116-8.

## Background

The high prevalence of childhood overweight and obesity [1] is considered a complex public health problem as it is driven by multiple, dynamic and interrelated factors, ranging from individual behaviours (e.g. daily sugar intake) to more upstream factors (e.g. urbanization). To address this complexity, systems approaches are increasingly being used in the development and implementation of public health programmes [2]. One such approach is system dynamics (SD), which possesses various characteristics, including programmes sensitive to starting conditions (context-specific); dynamic and adapting over time on the basis of new (system) insights; and developed through participatory processes [3, 4].

As SD approaches are sensitive to starting conditions and acknowledge that changes in initial conditions may influence programme effects, programmes should start with understanding the targeted system’s complexity [5]. In our case, this implies understanding how the current system contributes to childhood overweight and obesity prevalence. In public health research, this system understanding has mostly been operationalized through the development of causal loop diagrams (CLDs), which pose a visual representation of a system consisting of closed loops of causal influences. These CLDs are based on, for example, literature reviews, experts’ knowledge and group model building (GMB) workshops with the target group [6].

While CLDs are increasingly applied to demonstrate the complexity of public health problems, there are few examples of how such a system understanding can subsequently facilitate systems changes [6]. Systems changes can be facilitated by identifying and intervening on leverage points (LPs). LPs refer to places in the system where one can intervene to produce change across system parts and/or the system as a whole [7, 8]. Allegedly, the more LPs are targeted and the more diverse they are, the higher the chance of successfully facilitating systems changes [8]. A review on CLD development and application within public health found 12 out of 23 studies that identified LPs [6]. However, most studies only mentioned LPs as an aspirational next step to examine. Only three studies provided a more thorough description of LPs (concerning children’s environmental health [9], the coronavirus disease 2019 (COVID-19) pandemic [10] and policies addressing obesity [11]). None of the studies specified how LPs informed action programme development.

In theory, following the above-mentioned SD principles, programmes should target numerous LPs at different system levels, including the higher system levels. According to the framework for systems change by Foster-Fishman and colleagues [5], programmes must target both the system structure (which includes factors, connections and feedback loops) and function (which determines the system behaviour) to alter the status quo. For example, a particular programme advocates that the purpose of supermarkets should not only be to maximize profit for their shareholders (the current system function), but also to contribute to ‘raising’ healthy adolescents (the new system function). Although several frameworks exist that help distinguish the different system levels [7, 12–14], to the best of our knowledge, no study within public health illustrates how a system understanding can help identify LPs that can subsequently inform action development and contribute towards achieving systems changes.

In this paper, we used the Lifestyle Innovations Based on Youth Knowledge and Experience (LIKE) programme as a case study to illustrate how a previously obtained understanding of the pre-existing system of obesity-related behaviours in adolescents [15] was used to identify LPs and subsequently develop an action programme within a SD approach to inform and facilitate systems changes.

## Methods

### The LIKE programme

LIKE is part of the Amsterdam Healthy Weight Programme (AHWP), a local-government-led whole systems approach with the long-term goal of reducing childhood overweight and obesity in Amsterdam, the Netherlands [16]. LIKE focuses on the transition from child to adolescent (ages 10–14) and is implemented in three ethnically diverse neighbourhoods with a lower socioeconomic position in the Amsterdam East district. LIKE uses a SD and participatory action research approach in developing, implementing and evaluating a dynamic action programme that can help change the current system towards one where healthy lifestyle behaviours are promoted [17]. The LIKE consortium is a transdisciplinary team consisting of academic researchers, policymakers at the city level and Amsterdam East district and professionals working for the AHWP.

**Fig. 1:**
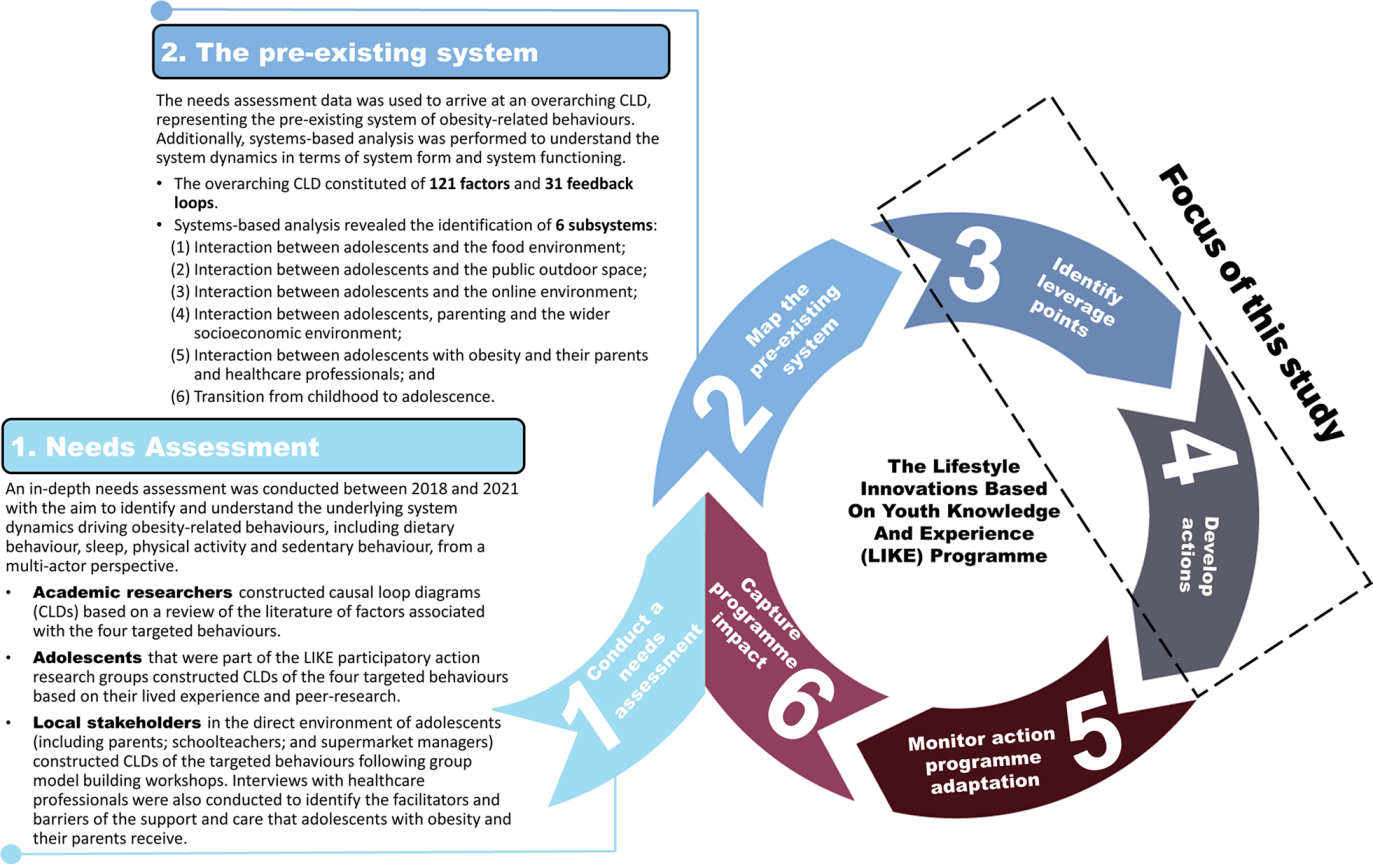
Overview of the LIKE programme

The LIKE programme centres around a six-stage cyclic process, including: (1) conduct a needs assessment; (2) map the pre-existing system; (3) identify LPs; (4) develop actions; (5) monitor action programme adaptation; and (6) capture programme impact (Fig. 1) [3]. Stages 1 and 2 took place between 2018 and 2021 and involved an in-depth mixed-methods needs assessment [17, 18]. This needs assessment captured an understanding of the underlying SD driving obesity-related behaviours from the perspective of multiple actors, including academic researchers, adolescents and local stakeholders, into a CLD. This CLD contained 121 factors and 31 feedback loops and consisted of six subsystems, including: (S1) interaction between adolescents and the food environment; (S2) interaction between adolescents and the public outdoor space; (S3) interaction between adolescents and the online environment; (S4) interaction between adolescents, parenting and the wider socioeconomic environment; (S5) interaction between adolescents with obesity and their parents and healthcare professionals; and (S6) transition from childhood to adolescence [15].

The current study builds on these system insights to develop a participatory SD action programme by identifying LPs (stage 3) that inform the development of actions (stage 4). In LIKE, adolescents and local stakeholders developed various action ideas using participatory action research (Emke et al., in preparation, 2024) and GMB (Waterlander et al., in preparation, 2024), respectively. In parallel, the LIKE consortium initiated additional action ideas by building on the insights gained from the action development process by adolescents and local stakeholders; targeting the functioning of the system; and conducting systems-based analysis. These consortium-initiated actions are the focus of the present paper.

Both stage 5 (monitor action programme adaptation) and stage 6 (capture programme impact) will be addressed elsewhere (de Pooter et al., in preparation, 2024; Luna Pinzon et al., in preparation, 2024). Furthermore, the current paper will not assess implementation, outputs and outcomes of the action programme. The remaining part of the methods section illustrates how the LIKE evaluation team (WW, ALP, NdP, KS) guided the LIKE consortium through a series of steps to identify LPs and develop action ideas. This study was approved by the institutional medical ethics committee of Amsterdam UMC, Location VUMC (2018.234).

### Procedure for the identification of leverage points and development of action ideas

The evaluation team guided the LIKE consortium through six steps to identify LPs and subsequently develop action ideas. These steps are outlined below in more detail.

#### Step 1: identifying underlying mechanisms

Step 1 involved determining which SD within the pre-existing system the LIKE consortium aimed to target (first). In February 2020, the LIKE consortium discussed all the collected data as part of the needs assessment stage thus far, focusing on the produced CLDs supplemented with data from the participatory action research groups and an overview of actions already taking place in the Amsterdam East district. All this information was collectively discussed with the aim to identify and prioritize underlying mechanisms, that is, a segment of a larger process in the system (causes of the causes) [18, 19], by asking the question: Taking into account the needs assessment results, what are the most important mechanisms contributing to unhealthy lifestyles among adolescents aged 10–14? Mechanisms were prioritized by considering system boundaries, which define the system parts that are included or excluded for this particular analysis [3]. These boundaries related to, for example, the focus on the transition period from childhood to adolescence and Amsterdam East as the setting.

#### Step 2: action-groups formation

In step 2, the LIKE consortium split up into groups to work on the identified mechanisms. Participants could join one or multiple groups on the basis of their expertise and/or interest. This resulted in the formation of five action-groups. A prerequisite was for each group to include at least two academic researchers, one professional working for the AHWP and one policymaker working for the municipality. Action-groups were encouraged to meet regularly to discuss their plan of action and plenary meetings with all action-groups were organized by the evaluation team every 6 weeks to discuss progress.

#### Step 3: further refinement of the identified mechanisms

In step 3, the evaluation team guided action-groups in understanding the targeted mechanisms from an SD perspective. Each group received an action-group workbook [see Additional file 1] with different sections to complete. These sections included: a description of the mechanism based on academic literature; an assessment of why the mechanism was relevant to the transition from child to adolescent; and an assessment about why the mechanism was particularly relevant at this moment (in comparison with, for example, 20 years ago). Action-groups were also encouraged to consult external experts to further refine their mechanisms.

#### Step 4: identification of leverage points and system levels analysis

Step 4 aimed to identify LPs that would help disrupt the identified mechanisms. To achieve this, we conducted systems-level analysis by applying the Intervention Level Framework (ILF). The ILF was developed by Johnston and colleagues to assist in finding solutions to complex health problems [13]. The ILF consists of five system levels and intervening at the higher levels will produce the most disruptive systems changes. The highest ILF level is a system’s paradigm, representing its deepest-held belief. Level two, the system, together with the system paradigm, dictates the way in which the system behaves and determines which system outcomes are produced. Level three is the system structure and defines the interconnections between the different system parts. Level four describes the system’s feedback and delays. This level refers to a system’s ability for self-regulation by supplying information about outcomes of actions back to the source of those actions. Lastly, level five describes the structural elements of a system in terms of actors or physical elements [13].

The action-groups used a table explaining the ILF levels and conducted ILF analysis. This analysis involved the identification of LPs for their mechanisms and the assignment of one of the five ILF levels to each LP. To facilitate this, action-groups used guiding questions, such as those described in the Action Scales Model [14]. For example, the following question helped in identifying a LP at the system paradigm level: What are the prevailing assumptions, beliefs and values that explain why things are done as they are? [14] LPs and their corresponding ILF levels were included in the action-group’s workbook [see Additional file 1].

#### Step 5: generating action ideas

In step five, action-groups generated action ideas on the basis of the identified LPs. At the action idea level, it was important for groups not to focus on the specific form of the action (e.g. a workshop) but to specify a theory of change in terms of the action function [3]. In other words, the theory of change specified how the particular action would target the identified LP, thereby contributing to disrupting the targeted mechanism and thus ultimately aiding in achieving the desired systems changes. To facilitate this, action-groups answered the question: Which actions can help target the LP and ultimately aid in achieving systems changes (define action idea in terms of action function and using the S.M.A.R.T criteria [20])? Action ideas were added to the action-group’s workbook [see Additional file 1] and included these characteristics: targeted mechanism and LP, system level (ILF level), action name, action form and action theory of change.

#### Step 6: assessing the degree to which action ideas could be embedded in existing initiatives

In step six, action-groups investigated which actions were already happening in the LIKE focus area to determine the degree to which action ideas could be embedded in existing initiatives. Action-group members working for the AHWP and municipality provided this information. Lastly, action-groups were encouraged to involve external stakeholders to aid in the further development of the action ideas.

### Reflection, adaptation and monitoring

Action development continuously occurred in a cyclic process, wherein ideas were adapted on the basis of the context and setting in which they ought to be implemented, as well as the feedback received from the evaluation team. For example, during action development, if it became apparent that an action idea was not feasible due to a lack of alignment with existing initiatives or redundancy, the idea was either adapted or abandoned. Similarly, efforts to emphasize actions at specific system levels were adjusted over time. For instance, the focus on targeting higher system levels was increased when we observed a shortage of action ideas at those levels. The evaluation team supported action-groups in applying systems thinking throughout the action programme development process via developed workbooks (see Additional file 1), the use of guiding questions and by organizing plenary meetings. To track the progress of the action programme, a monitoring system was set up, composed of: action-group’s workbooks; action register database containing, for example, name, action form and function; and stakeholder database containing the type of stakeholders involved.

### Data sources and analysis

For data analysis, the lead researcher (ALP) read and summarized all action-group workbooks and extracted action ideas generated by the LIKE consortium from the action register database. A second researcher (NdP) supported this process. Note that action-groups generated various action ideas throughout the process, and that not all actions were actually executed or specified into detailed action plans. This paper focuses on all actions for which action-groups provided specified theories of change. Identified mechanisms, targeted LPs and corresponding action ideas were ordered per subsystem. The evaluation team discussed preliminary findings to ensure that these reflected the process followed within the LIKE programme.

## Results

Five of the six previously identified subsystems (stages 1 and 2 [15]) were targeted by the action-groups. These included: food environment, public outdoor spaces, socioeconomic environment, healthcare and transition from child to adolescent. No LPs specifically targeted subsystem 3, describing the interaction between adolescents and the online environment as action-groups prioritized the other subsystems. The remaining part of this section does not discuss this subsystem.

**Fig. 2:**
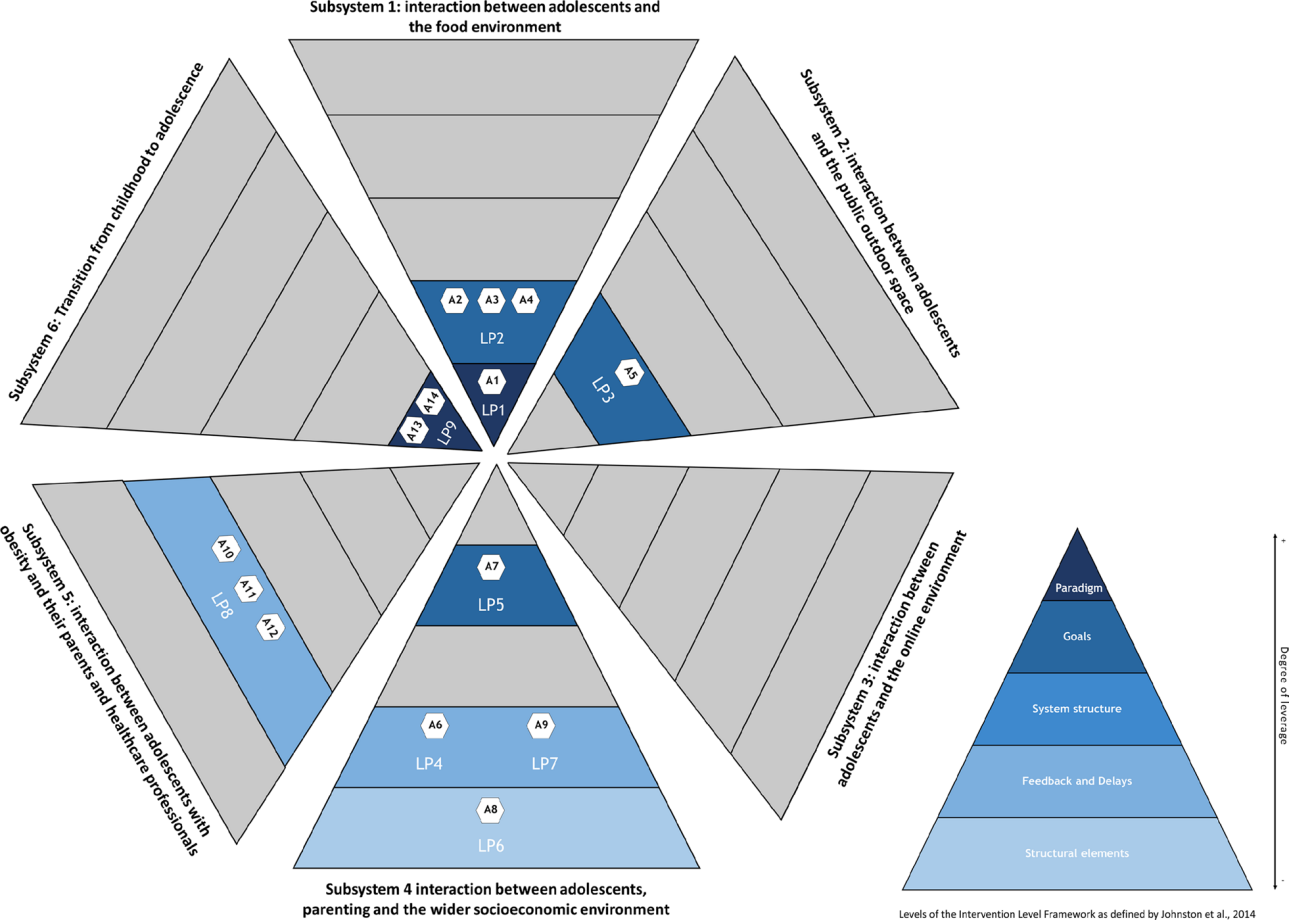
Overview of targeted subsystems, identified leverage points (LPs) and developed action ideas (A)

Within the five targeted subsystems, action-groups initially identified 12 mechanisms. After prioritizing, we selected eight mechanisms to further identify LPs and subsequently develop action ideas. These final mechanisms included: (M1) power dynamics in the current food system; (M2) the use of public outdoor spaces for physical activity by adolescents; (M3) the role of parents during adolescence; (M4) livelihood security and poverty; (M5) connection between health ambassadors (volunteers), municipality and community organizations; (M6) match between local health promotion activities and parents’ needs; (M7) match between obesity healthcare services and the needs of adolescents with obesity and their parents; and (M8) social norms influencing health behaviours in adolescents. Within these mechanisms, action-groups initially defined a wide range of potential LPs at each of the ILF levels. After refinement, action-groups made a final selection of 9 LPs, from which they developed a total of 14 action ideas. Figure 2 provides a graphical representation of how these action ideas were divided across the LPs and which of the five ILF system levels these LPs targeted (referred to as LP1–LP9 in Fig. 2). We describe these results per subsystem below.

### Subsystem 1 regarding the interaction between adolescents and the food environment: mechanisms, leverage points and action ideas

A total of one mechanism and two leverage points were identified and four action ideas developed within subsystem 1 (Table 1). M1 refers to the power dynamics that exist between local food retailers that want to offer healthier food products and large global food companies selling unhealthy food products for attractive profits. To arrive at the final LPs selection, action groups used two guiding questions: Who are the key decision-makers in shaping the local food environment for adolescents? and, Given our established networks within the local and national food system, how can we use our sphere of influence to make the Amsterdam food system healthier?

**Table 1:** Subsystem 1 regarding the food environment: mechanisms, leverage points and action ideas

Mechanism	Description	Leverage point	System level (ILF)	Action idea name	Form of action idea	Action idea theory of change	External stakeholders involved
(M1) Power dynamics in the current food system	In the current food subsystem, a power imbalance exists between large global food companies and smaller local companies. This imbalance results from the large profits made by global food companies on unhealthy food sales, which are more profitable than healthier options. These profits can in turn be further invested in, for example, marketing and lobbying, thereby further boosting the demand for unhealthy food Smaller local companies, which may offer healthier food, can therefore hardly compete against the economic and political power of these large food companies	(LP1) Supermarkets and schools take joint responsibility for the role they play in shaping adolescents’ food environment	2 – Goals	(A1) GMB workshops with food retailers and/or schools	Group model building sessions with food retailers and/or schools	Supermarkets do not generally feel that adolescents’ eating behaviour is also their responsibility. By coming together to discuss the role they play in shaping the local food environment of adolescents, supermarkets will own their responsibility in shaping adolescents’ food environment and will be encouraged to come up with actions that limit their unhealthy food supply	Supermarkets; schools
		(LP2) Policies that increase and support the availability and accessibility of healthy food	2 – Goals	(A2) Exposing retails tactics and lack of action in obesity prevention	Interviews with supermarket managers and marketing experts with the aim of understanding their operating space to become healthier	Many local interventions have tried to make supermarkets a healthier environment for adolescents. Most of these interventions have, however, only show limited impact, and if successful, are terminated because they result in profit losses. By synthesizing evidence about the lack of impact of these local interventions, we hope to build an evidence base that shows the need for policy intervention rather than self-regulation	Supermarkets
				(A3) Developing an active lobbying initiative between academia and policy practice	Monthly meetings between LIKE researchers and the municipality to discuss and plan strategies that influence the local and national food policies	Meetings between academia and policy provides insights into each other’s worlds and helps in gaining lobbying power towards national policy. For example, local Amsterdam policymakers know where in the policy cycle national health policies are, and can come up with a strategic plan based on local evidence that we can generate to influence this policy process	N.A.
				(A4) Entrepreneur network	Monthly meetings between LIKE researchers and the manager of the municipality’s entrepreneur network to exchange knowledge	Many small entrepreneurs in Amsterdam East sell healthy food but are forced to also offer unhealthy food to keep financially afloat. This is because selling unhealthy food is much more lucrative. If local entrepreneurs can be structurally better supported in offering healthier food, they can counterbalance the power of larger food companies such as supermarkets and fast food chains. This could be achieved through, for example, policy changes that help increase the supply and accessibility of healthy food	N.A.

The first guiding question resulted in the identification of supermarkets as one of the most important players in shaping the food environment. ILF analysis revealed that, ultimately, changes in system goals would be needed to create healthier supermarkets. Otherwise, the system reverts to generating solely financial profits (LP1). A1 (GMB workshops) therefore aimed to change the beliefs of local supermarkets by involving them in GMB workshops. During these workshops, supermarkets would analyse the food system, thereby highlighting their role in shaping it.

The second guiding question identified the food policy context (national and local) as an important factor to influence (LP2). Hence, A2 (exposing retails tactics and lack of action) aimed to generate local evidence about the need for top-down measures from the government. A3 (active lobbying initiative) and A4 (entrepreneur network) focussed on improving and maintaining the collaboration between academia and the municipality. Their goal was to exchange knowledge and information, thereby contributing to a shared agenda.

### Subsystem 2 regarding the interaction between adolescents and the public outdoor space: mechanisms, leverage points and action ideas

One mechanism and one leverage point were identified and one action idea developed that targeted subsystem 2 (Table 2). M2 outlines how the increasing density in cities such as Amsterdam has resulted in limited public outdoor spaces for active play and sports, as well as unattractive spaces for adolescents. Moreover, although citizen participation is gaining popularity, participation of adolescents in the design of public outdoor spaces is not yet common in policy practice. ILF analysis revealed that to disrupt M2, a new system goal was required, prioritizing the redesign of public outdoor spaces in co-creation with adolescents. The goal is to make these spaces more attractive, especially for active play and sport (LP3).

**Table 2:** Subsystem 2 regarding the public outdoor space: mechanisms, leverage points and action ideas

Mechanism	Description	Leverage point	System level (ILF)	Action idea name	Form of action idea	Action idea theory of change	External stakeholders involved
(M2) The use of public outdoor spaces for physical activity by adolescents	In highly populated cities such as Amsterdam, the high demand for housing and business has resulted in public outdoor spaces designed for active play and sports being built on the outskirts of neighbourhoods. Larger distances to such spaces are a barrier for adolescents. Furthermore, public outdoor spaces are generally unattractive for adolescents as they are often designed for younger children or adults or for one specific activity, for example, basketball or soccer. Furthermore, adolescents are not engaged in the decision-making /design/organization of public outdoor spaces, resulting in these spaces not matching their wishes and/or needs	(LP3) Adolescents participate in decision-making about the design and organization of public outdoor spaces	2 – Goals	(A5) Co-creation of the public outdoor space	Organize a project where adolescents, the municipality and community organizations co-create the design of a public outdoor space with the aim of making it more attractive for outdoor active play and sport from the adolescent perspective	By co-designing a public space in the neighbourhood with adolescents, experiences about the co-creation process can be gathered and shared with relevant actors. Positive experiences with co-creation will increase the support among policymakers for actively engaging adolescents in decision-making about the design of public outdoor spaces. This might ultimately increase the attractiveness of these spaces, thereby promoting adolescents’ physical activity levels	Community organization focused on youth participation and participatory youth research; schools

The guiding question used was: How can we use our experience with co-creation to alter the public outdoor space in such a way that adolescents make more and active use of it? A5 (co-creation outdoor space) therefore aimed to organize a co-creation process wherein adolescents, the municipality and community organizations participated in redesigning a designated outdoor space close to a local school. The insights gained could then serve as a lever to advocate for the inclusion of adolescents in the future design of public outdoor spaces.

### Subsystem 4 regarding the interaction between adolescents, parenting and the wider socioeconomic environment: mechanisms, leverage points and action ideas

A total of four mechanisms and four leverage points were identified and four action ideas developed that targeted subsystem 4 (Table 3).

**Table 3:** Subsystem 4 regarding the wider socioeconomic environment: mechanisms, leverage points and action ideas

Mechanism	Description	Leverage point	System level (ILF)	Action idea name	Form of action idea	Action idea theory of change	External stakeholders involved
(M3) The role of parents during adolescence	Parents undergo a new role from a managerial to a more coaching role of their children in the transition from child to adolescent. Because of this, they may find it difficult to set, monitor and enforce rules regarding sleep, dietary behaviour, screen behaviour and physical activity. Adolescents have difficulties making sensible choices regarding bed time and screen use in the evenings and indicate that they need their parents to set rules	(LP4) Parents can set, monitor and enforce rules regarding sleep, dietary, screen and physical activity behaviour	4 – Feedback and delay	(A6) Rules Rule	Workshop in which parents who serve as health ambassadors in the neighbourhood learn about parenting skills from a professional and from each other and how to set and enforce rules. The insights gathered during the workshop will be transferred to short vlogs which will be disseminated to other parents	Health ambassadors indicated that they need more information about setting and enforcing rules to disseminate to other parents. By educating health ambassadors about parenting skills around healthy behaviours, they will share this knowledge with fellow parents in the neighbourhood and contribute to structurally set and enforce rules that support healthy behaviours	Social welfare organizations focused on helping community members to actively participate in society and receive community support
(M4) Livelihood security and poverty	When families live in relative poverty, the problems and stress they experience may occupy parents’ headspace. As a result, they often pay less attention to stimulating healthy behaviours in their children	(LP5) Health is included as an important topic in policies that relate to social security	2 – Goals	(A7) Connecting health and livelihood security	Investigate the possibilities to connect the three policy areas of the municipality that address livelihood security: household income, housing and health	Integrating household income, housing and health in policies that aim to improve social security may offer a more effective approach to diminish the number of families living in poverty and thereby contribute to better living conditions and healthier behaviours for adolescents	N.A.
(M5) Connection between health ambassadors (volunteers), municipality and community organizations	In the LIKE focus area, community health ambassadors, who are mostly parents, play an important role in stimulating local residents (such as fellow parents) towards developing healthier habits. However, the support that health ambassadors receive from the municipality and community organizations does not sufficiently match their wishes and expectations. This results in less and less health ambassadors committing to influencing local residents towards a healthier lifestyle	(LP6) Improve the commitment of health ambassadors to influence local residents towards a healthy lifestyle	5 – Structural elements	(A8) Interviews with health ambassadors about their role	Interview health ambassadors about their experiences, needs and wishes related to their role as health ambassadors. Next, write a recommendation to the municipality and community organizations about the optimal support of health ambassadors	By gaining insight into the differences in perspectives between health ambassadors and community organizations, these insights can be implemented in practice, thereby improving the support health ambassadors receive	Social welfare organizations focused on helping community members to actively participate in society and receive community support
(M6) Match between local health promotion activities and parents’ needs	In the LIKE focus area, there are many health promotion activities organized for parents. However, there is a mismatch between the content and type of such activities and the needs and wishes of parents Because of this, professionals organizing these activities and parents attending such activities have problems in communicating and understanding each other. This in turn can lead to demotivation, misunderstanding and uncertainty among both parents and professionals organizing the health promotion activities, thereby forming a barrier to the promotion of a healthier lifestyle	(LP7) Local health promotion activities meet parents’ needs	4 – Feedback and delay	(A9) Case study health promotion activity called ‘parenting debates’	Conduct interviews with participants, debate leaders and project designers of the parenting debates that are organized by a community organization and focus on creating a healthy lifestyle for families	Some parents, especially fathers, may be difficult to reach by interventions that aim to improve parenting skills around a healthy lifestyle. By gaining insights about an intervention offered by a community organization that organizes group discussions and is able to successfully reach fathers, these insights can be used to engage these parents in other interventions and programmes that also aim to encourage a healthier lifestyle	Community organization that organizes parenting debates; youth healthcare services

In the transition from child to adolescent, parents undergo a new role from a more managerial to a more coaching role of their children (M3). Parents might therefore find it difficult to set, monitor and enforce rules regarding healthy behaviours (LP4). The guiding question used was: Which initiatives already exist that reach parents and can help improve parental skills? This resulted in the identification of a programme within the AHWP, where parents take the role of health ambassadors to stimulate fellow parents to encourage a healthier lifestyle in their children. A6 (Rules Rule) aimed to educate health ambassadors about parenting skills so that they can further spread this knowledge with other parents in the community.

Subsystem 4 further relates to households living in relative poverty in Amsterdam East, whereby financial and social problems may accumulate, resulting in higher stress levels and less attention for creating and sustaining healthy behaviours amongst parents (M4). ILF analysis revealed that a new system goal was needed in which parents would no longer be forced to prioritize household livelihood security at the expense of stimulating healthy behaviours (LP5). The guiding question used was: Which municipal policy systems have overlapping goals and to what extent can these be aligned? This resulted in A7 (connecting health and livelihood security), which aims to investigate how the municipality can avoid working in silos and integrate the three policy areas of household income, housing and health.

The last two mechanisms identified relate to common misunderstandings between professionals and parents in the work of the health ambassadors (M5) and in the local health promotion activities offered to parents (M6). ILF analysis revealed that health ambassadors do not feel supported in their work, which negatively influences their commitment (LP6). A8 (interviews with health ambassadors) therefore focuses on addressing this perceived lack of support. ILF analysis further revealed that current health promotion activities do not match the expectations and needs of parents (LP7). A9 (parenting debates) thus aims to gain a better understanding of which factors contribute towards matching the needs of parents so that these insights can be disseminated to other activities.

### Subsystem 5 regarding the interaction between adolescents with obesity and their parents and healthcare professionals: mechanisms, leverage points and action ideas

One mechanism and one leverage point were identified, and three action ideas developed that targeted subsystem 5 (Table 4). Mechanism 7 describes that the working methods, organization and competences of healthcare professionals do not sufficiently fit the needs of adolescents with obesity and their parents. The national model for integrated care for childhood overweight and obesity [21–23] encourages healthcare professionals to take the complexity embedded in factors related to childhood obesity into account in the support and care systems. However, there are barriers in the implementation of the model, for example, a lack of time and resources within the current healthcare system, and the need to invest in a strong family–professional relationship [24, 25].

**Table 4:** Subsystem 5 regarding healthcare: mechanisms, leverage points and action ideas

Mechanism	Description	Leverage point	System level (ILF)	Action idea name	Form of action idea	Action idea theory of change	External stakeholders involved
(M7) Match between obesity healthcare services and the needs of adolescents with obesity and their parents	The working methods, organization and competences of healthcare professionals do not sufficiently fit the needs and possibilities of the multi-ethnic target group in Amsterdam East	(LP8) Increase the experienced support and effectiveness of the obesity care received by families from healthcare professionals	4 – Feedback and delay	(A10) Organization-wide scan to identify to what extent the youth healthcare system is sensitive to ethnic diversity	Conduct an organization-wide assessment to determine the degree of diversity-responsiveness of the youth healthcare services and use this information to develop an action plan	If the working methods, organization and competences of youth healthcare professionals are more in line with the expectations and characteristics of the multi-ethnic target group in Amsterdam East, families could experience more support with regard to the obesity care they receive	Youth healthcare services
				(A11) Examination room observations	Observe the communication between healthcare professionals and families in the examination room. Next, provide feedback to the healthcare professional about the communication and disseminate the learned lessons to healthcare professionals	The expectations of families and healthcare professionals do not always match. This results in families having a negative experience with regard to obesity care, making it difficult for families to improve their lifestyle. One of the contributors to this mismatch in expectations is suboptimal communication between families and healthcare professionals. By increasing awareness amongst healthcare professionals about communication with children with obesity and their parents, guidelines in obesity healthcare regarding communication can be improved. This could result in healthcare professional–family communication that matches the family’s expectation	Childhood obesity healthcare
				(A12) Popular scientific article about motivation	Interview healthcare professionals about their perspective on the motivation of children with obesity and their parents with regard to lifestyle changes. Next, write popular scientific article about the insights obtained from the professionals. And lastly, actively disseminate those insights amongst healthcare professionals	While many healthcare professionals are aware that obesity is caused by many factors besides individual factors, the family’s motivation is often mentioned as an important barrier for behaviour change. Increasing awareness amongst healthcare professionals around this topic could influence the attitude of healthcare professionals towards the family	Childhood obesity healthcare

ILF analyses indicated that to help disrupt M7, the support and care families receive from healthcare professionals needed intensifying (LP8). A10–12 therefore aim to help tailor the childhood obesity support and care to the needs of adolescents with obesity and their parents with the goal of empowering families to achieve a healthier lifestyle.

### Subsystem 6 regarding the transition from childhood to adolescence: mechanisms, leverage points and action ideas

One mechanism and one leverage point were identified and two action ideas developed that targeted subsystem 6 (Table 5). M8 describes how adolescents consider unhealthy behaviours normal and cool as part of the social norm. As adolescents seek acceptance from their peers, they will, therefore, not deliberately deviate from this social norm.

**Table 5:** Subsystem 6 regarding the transition from childhood to adolescence: mechanisms, leverage points and action ideas

Mechanism	Description	Leverage point	System level (ILF)	Action idea name	Form of action idea	Action idea theory of change	External stakeholders involved
(M8) Social norms influencing health behaviours in adolescents	Health behaviours are strongly influenced by social norms in a specific group, and that behaviour in turn influences the social norm. During the transition from childhood to adolescence, adolescents typically desire to be part of and accepted by a group, thereby making them extra vulnerable to the influence of, for example, peers and friends. Adolescents usually exhibit unhealthy behaviours when hanging out with friends. The current social norm amongst adolescents is that unhealthy behaviour is cool	(LP9) A new social norm in which exhibiting healthy behaviours is considered cool and normal	1 – Paradigm	(A13) Peer role models	Implement and evaluate the use of peer role models in a local community organization that stimulates healthier behaviours among adolescents	By synthesizing insights about the effect of using peer role models in a local project that stimulates healthier behaviours in adolescents, and sharing these insights with community organizations that run similar projects in Amsterdam East, these organizations will also be encouraged to use peer role models to stimulate healthier behaviours in adolescents. As a result, more adolescents will show healthier behaviours at the activities organized by the community organizations, which will help change the social norm towards healthy behaviours as cool and normal	Community organization that focuses on stimulating healthier behaviours among adolescents
				(A14) Role models network of youth workers and adolescents	Create a network of youth workers and adolescents that commit to becoming role models for stimulating healthier behaviours. Organize sessions to discuss what is needed to influence adolescents towards exhibiting healthier behaviours and provide training and/or materials	By creating a role model network, youth workers and adolescents will develop a shared vision and communication strategy about how to stimulate healthier behaviours among their peers. These strategies can then be implemented in activities organized for adolescents in Amsterdam East, thereby encouraging a new social norm towards healthier behaviours	Community organizations working with youth workers and adolescents

ILF analysis revealed that to help disrupt M8, the social norm of exhibiting unhealthy behaviours (especially when hanging out with peers) needed to be altered (LP9). One way to initiate this shift in belief is by using role models to encourage adolescents to start exhibiting healthier behaviours. A13 (peer role models) and A14 (role models network) aim to use peers and youth workers as agents for changing the unhealthy social norm (A13–A14).

## Discussion

### Principal findings

This study presents the outline of an action programme tackling obesity-related behaviours in the transition period from childhood to adolescence (ages 10–14) within an SD approach. We developed the action programme by translating a previously obtained system understanding into mechanisms and subsequently identifying LPs and developing action ideas that can contribute towards achieving system changes. Interdisciplinary action-groups were formed, which were actively guided in applying systems thinking throughout the development process. The resulting action programme focussed on 8 mechanisms using 9 LPs and 14 action ideas with aligning theories of change targeting both the system’s structure and function. This paper thereby illustrates the feasibility of formulating actions targeting higher system levels within the confines of a research project timeframe when sufficient and dedicated effort is put into this process.

### Comparison with the development of other system approaches

Substantial heterogeneity exists in public health programmes that take a system’s approach in terms of design, implementation and outcomes produced [2]. Within public health, GMB is the most commonly used method to gain system understanding and/or inform the development of actions [6]. In the context of childhood overweight and obesity prevention, most examples on the use of GMB to develop actions can be found in programmes conducted in Australia [26–28].

One such example is the Whole of Systems Trial of Prevention Strategies for Childhood Obesity conducted in Victoria, Australia [26]. In this programme, a system understanding was developed by combining anthropometric and local behavioural data with CLDs produced in GMB sessions with community members. From these sessions, an action programme with more than 400 actions was created. Unfortunately, details of the process following the move from system understanding to a SD action programme are lacking. The authors do mention that a similar framework to the ILF was applied (Foster-Fishman’s framework for transformative systems change [5]) to retrospectively gain insights into the system levels that actions were targeting. However, the application of this framework was conducted independently from the action development process by the communities [29]. Details about how SD actions were constructed prospectively are important because it can help guide other programmes taking a SD approach.

Another study also used community GMB workshops to identify systemic barriers to fruit and vegetable intake in children in New Zealand [30]. Study participants developed actions by taking into account LPs and answering three questions: What variables (of the CLD) could you increase or decrease?; How could you impact connections: strengthen, or weaken a connection, speed it up or slow it down, add or delete connections?; and How could you impact the ‘rules’ that govern the system or the goals that it is trying to achieve? [31]. This resulted in 18 actions that targeted four subsystems, including the home environment, fast food, community nutrition and health outcomes. The authors, however, concluded that participants were unable to generate specific action ideas that took into account the higher system levels and instead reverted to traditional, individual-focused actions. They also concluded that this was likely due to insufficient time being allocated for this process [30]. However, it could also be the case that guiding questions alone do not sufficiently aid in arriving at the higher system levels and that the application of a framework, such as the ILF, is also needed. In LIKE, we tried to overcome these issues by guiding action-groups in the whole process from system understanding to action development. Furthermore, we allocated sufficient time and guidance for groups to familiarize themselves with the concept of applying the ILF to identify LPs through practical exercises and to allow actions to adapt over time as system insights increased.

### Reflections on the methods followed to identify leverage points and develop action ideas

In SD approaches, it is crucial to develop an a priori system understanding because this serves as the starting point to identify LPs and develop actions. In addition, it is crucial that this system understanding is shared among stakeholders who are involved in the development of actions and who may not necessarily be involved in the previous phases. This shared understanding will contribute to developing a shared vision of what the programme aims to achieve and thereby help promote ownership of the problem and potential solutions [5]. SD approaches are typically formed by transdisciplinary teams who work together to understand and change the targeted system [32–34]. However, the challenges associated with how such teams are expected to work in practice are underreported [34]. The LIKE consortium included representatives from academia, policy and practice. To promote cohesive teamwork, a shared vision, trust and commitment within the project, substantial efforts were made throughout the duration of LIKE. These efforts included, amongst other things, regular meetings (four times per year) of the LIKE consortium to determine, for instance, the content of the action programme being developed and a workshop focused on clarifying the roles and responsibilities of the different LIKE members. Further details about the process of collaborating with different system actors when developing and implementing a SD action programme will be described elsewhere (Luna Pinzon et al., in preparation, 2024).

One way to support the identification of LPs beyond the qualitative process described in this paper is with the use of quantitative SD models. These models can explore potential futures and ask ‘what if’ questions [7]. In the context of the LIKE programme, this would entail translating the pre-existing CLD of obesity-related behaviours into a SD model, for example, using the methodology described in Crielaard et al., 2022 [35]. Next, it can, for example, be tested whether LP X would be a more promising lever to change the system instead of LP Y. On the basis of these ‘what if’ scenarios, a selection can be made as to which LPs to focus on. However, these models require data that can represent the factors included in the CLD, which we do not have access to in LIKE. Results from the wider LIKE evaluation could possibly be used to develop these models in the future.

The application of the ILF in this study was challenging due to its theoretical nature and the requirement of expertise on systems thinking [13, 14]. We tried to overcome this challenge by establishing an evaluation team that supported action-groups throughout the process with workbooks, using guiding questions and organizing plenary meetings. Recently, other frameworks to identify LPs have been developed, in addition to the ILF. First, Nobles et al. [14] developed the Action Scales Model, which also expands upon Meadows’ original 12 places to intervene in a system [7]. At the time LPs were identified in LIKE, the Action Scales Model had not yet been developed and therefore only the guiding questions of the action scales model to identify LPs were used in this study to supplement our ILF procedure. Another recently developed alternative to the ILF is the Public Health 12 framework [12]. This framework offers a translation of Meadows’ original 12 levels into a language that is practical and enables the operationalization of systems changes within public health [12]. On the basis of the challenges we faced in LIKE with the application of the five broader levels of the ILF, we believe that the application of the Public Health 12 framework will only be possible once enough experience is gathered in correctly distinguishing each system level and understanding its corresponding LPs.

In this paper, we departed from a more ‘traditional’ view of public health prevention programmes or interventions as a ‘standardized package of actions’. Instead, we applied a SD perspective and arrived at a ‘SD action programme’. An important characteristic of such a SD action programme is that actions are not only defined in terms of their form, but also in terms of their function by making their theories of change explicit. Furthermore, by consciously differentiating and considering whether LPs and actions target the structural (lower) system levels or the more functional (higher) system levels, we found that we were gradually able to formulate actions targeting the higher system levels. Although we acknowledge that instigating ‘genuine’ changes in systems paradigms or goals requires more time than a typical research project allows, this paper illustrates the feasibility of formulating actions targeting higher system levels within the confines of a research project timeframe when sufficient and dedicated effort is put into this process.

An important prerequisite to note here is a certain level of flexibility amongst all stakeholders involved due to the dynamics nature of a systems approach. For example, as insights of the system emerge over time, actions would need to be potentially adapted or abandoned [3]. The challenges arising from this dynamic process (de Pooter et al., in preparation, 2024) as well as lessons learned from the process of implementing a system dynamics project into a real-life setting (Luna Pinzon et al., in preparation, 2024), will be thoroughly analysed in separate articles. This includes the importance of trust among project members that is needed to deal with the dynamic character of the programme, as well as the importance of combining expertise in systems thinking with an understanding of the context and environment in which these actions are implemented.

### Strengths and limitations

To the best of our knowledge, this is the first study that presents the development of a SD action programme informed by a previously obtained systems understanding and targeting multiple system levels. Furthermore, this study illustrates how LPs can be identified prospectively and how these can inform the development of actions that facilitate systems changes. This study brings important findings as to what an action programme within a SD approach could look like and how this differs from more traditional public health interventions. Nevertheless, while promising, our study does not provide evidence on the effectiveness of this action programme in terms of concrete system outcomes. Changing a system is a participatory process that can take up to several years and these types of evaluation questions will be answered in future studies.

In terms of the action development process, we initially aimed to develop a programme that was dynamic and could be adapted on the basis of the new insights that emerged from the system [3, 36]. Although this was achieved to some extent (details of the LIKE action programme adaptation will be described elsewhere), not all of this was possible because of the COVID-19 pandemic and lockdowns. This resulted in a loss of momentum for some of the action-groups, and online instead of physical meetings and information gathering.

Lastly, the results presented in this study constitute only a part of the LIKE action programme. The LIKE action programme is composed of a wider collection of mechanisms, LPs and actions which are developed using participatory action research with adolescents and GMB sessions with stakeholders. The results from these participatory processes will be described in detail elsewhere (de Pooter et al., in preparation, 2024).

## Conclusions

This study provides details on the development of a SD action programme targeting obesity-related behaviours in adolescents. Interdisciplinary action-groups were supported in the identification of system’s mechanisms and the application of the ILF to identify LPs targeting system structure and function. The results show how such a SD action programme differs from traditional public health interventions. This is achieved by describing action ideas in terms of function, theories of change and the system levels they are targeting, thereby demonstrating the feasibility of developing actions targeting higher system levels within the constraints of a research project timeframe. We believe these insights can contribute to the further development and implementation of SD approaches within public health.

### Supplementary Information

**Additional file 1.** Action-group workbook template. The additional table includes the template of the workbook used by action groups in the LIKE programme to identify leverage points and develop action ideas.

## Data Availability

The datasets supporting the conclusions of this article are included within the article and its additional file.

## Abbreviations

SD: System dynamics
CLD: Causal loop diagram
GMB: Group model building
LPs: Leverage points
ILF: Intervention level framework
