# Supplementary material for: Development of an action programme tackling obesity-related behaviours in adolescents: a participatory system dynamics approach
Source: Health Res Policy Syst. 2024 Mar 1;22:30. doi: 10.1186/s12961-024-01116-8 (PMC10908105; doi:10.1186/s12961-024-01116-8)
Supplement: Supplementary file 1 — Additional file 1. Action-group workbook template. The additional table includes the template of the workbook used by action groups in the LIKE programme to identify leverage points and develop action ideas. [file 12961_2024_1116_MOESM1_ESM.docx]

**Additional file 1: Action-group workbook template**

Action-group:

Mechanism name:

1. **Mechanism that contributes to an unhealthy lifestyle among 10-to-14 years old adolescents**

| 1. Provide a description of the targeted mechanism based on the academic literature: |
| --- |
|  |
| 1. Why is the targeted mechanism relevant to adolescents in the transition from child to adolescent? |
|  |
| 1. Why is the targeted mechanism particularly relevant now (in comparison to for example 20 years ago)? |
|  |

1. **How can you disrupt the targeted mechanism based on the Intervention Level Framework (ILF) levels?**

| **Five system levels based on the ILF** | | | | |
| --- | --- | --- | --- | --- |
| Paradigm | Goals | System structure | Feedback and delays | Structural elements |
| **What are potential leverage points (at each of the 5 ILF levels) for disrupting the targeted mechanism in order for the system to ultimately 'produce' healthy behaviours?** | | | | |
| *Which system paradigm needs to be shifted or which new system paradigm is needed?* | *Which system goals can help achieve the required system paradigm shift?* | *What changes must take place in the structure of the relevant system to achieve these goals?* | *How can monitoring and feedback positively strengthen the mechanism and how can we possibly built monitoring/evaluation activities for this?* | *What elements in the system can you address with actions that support a shift in the system’s paradigm?* |
|  |  |  |  |  |
| **Which actions can help target the leverage point and ultimately aid in achieving systems changes (define action idea in terms of action function and using the S.M.A.R.T criteria)** | | | | |
|  |  |  |  |  |
| **What does the AHWP or municipality already do to achieve this action function?** | | | | |
|  |  |  |  |  |
| **What can the AHWP or municipality additionally do to achieve this action function?** | | | | |
|  |  |  |  |  |
| **Which stakeholders or organizations should be involved in the further development and implementation of this action?** | | | | |
|  |  |  |  |  |
| **With which other mechanisms is this action associated?** | | | | |
|  |  |  |  |  |
| **Where do you see common ground with PAR / GMB / other LIKE findings?** | | | | |
|  |  |  |  |  |
